# Baduanjin exerts anti-diabetic and anti-depression effects by regulating the expression of mRNA, lncRNA, and circRNA

**DOI:** 10.1186/s13020-019-0225-1

**Published:** 2019-02-01

**Authors:** Tian An, Zhong-Chen He, Xin-Qing Zhang, Jun Li, Ai-Ling Chen, Fang Tan, Hong-Dong Chen, Bo-Han Lv, Juan Lian, Si-Hua Gao, Guang-Jian Jiang

**Affiliations:** 1 Diabetes Research Center, Traditional Chinese Medicine School, Beijing University of Chinese Medicine, Beijing, 100029 China; 2 Department of endocrinology, Beijing He ping li Hospital, Beijing, 100013 China; 3 Department of Neurosurgery, ChuiYangLiu Hospital affiliated to Tsinghua University, Beijing, 100022 China; 4 Chinese Medicine Department, ChuiYangLiu Hospital Affiliated To Tsinghua University, Beijing, 100022 China

**Keywords:** Baduanjin, Diabetes, Depression, CircRNAs, LncRNAs

## Abstract

**Background:**

Baduanjin, a traditional Chinese exercise therapy, has been widely used in China to treat type 2 diabetes (T2DM) with depression (DD). However, the underlying mechanism of Baduanjin in anti-DD is unclear. This study was focused on investigating the effects of Baduanjin on symptoms of depression and blood glucose in patients with DD and the underlying mechanism.

**Methods:**

We performed a 12-week Baduanjin intervention on patients with DD and longitudinally compared the differential expressions of lncRNAs, circRNAs, and mRNAs between pre- (BDD) and post- (ADD) Baduanjin intervention in the same group. Subsequently, Gene Ontology (GO) and pathway analysis was performed to investigate the function of differentially expressed mRNAs. Finally, Reverse Transcription-Polymerase Chain Reaction (RT-PCR) was used to verify the sequencing result and the mRNA-lncRNA regulatory network was constructed.

**Results:**

The blood glucose level, depression index scores, and PHQ9 scores of the patients with DD were significantly decreased (P < 0.05) after Baduanjin intervention. Compared to BDD, 207 lncRNAs, 266 circRNAs, and 610 differentially expressed mRNAs were identified in ADD. Kyoto Encyclopedia of Genes and Genomes (KEGG) and GO showed that the significantly dysregulated mRNAs were mainly involved in immune function and inflammatory response pathways, and various signaling pathways including IL-17 and TNF. In addition, we selected five differentially expressed lncRNAs to construct an lncRNA-mRNA regulatory network, and found a total of 1045 mRNAs associated with them.

**Conclusions:**

Our research is the first systematic profiling of mRNA, lncRNA, and circRNA in patients of ADD and BDD, and provides valuable insights in the potential mechanism of Baduanjin in anti-DD. Further, it was confirmed that Baduanjin is a safe and effective intervention for patients with DD because it can effectively ameliorate the symptoms of depression and blood glucose levels in patients with DD by regulating the dysregulated expression of lncRNA, mRNA, and circRNA.

**Electronic supplementary material:**

The online version of this article (10.1186/s13020-019-0225-1) contains supplementary material, which is available to authorized users.

## Background

Baduanjin (Eight-Section Brocades) is a traditional form of exercise that has been extensively practiced in China for many centuries [1]. Baduanjin contains eight simple actions based on Traditional Chinese Medicine (TCM) theory. The main focus is to assimilate energy in the body, which causes a variety of health benefits [2]. Baduanjin makes the posture and movement of the body symmetrical, and the movement of the mind and breathing become harmonious [3]. Due to the unique physical and mental relaxation, improved concentration, and better respiratory control it helps attain, it is suggested to improve Type 2 diabetes (T2DM) with symptoms of depression [4, 5]. Previous studies have shown that Baduanjin can relieve musculoskeletal pain, reduce anxiety and depression, and regulate glucose and lipid metabolism [6–9]. These studies provide a foundation for us to study Baduanjin as a potential non-drug anti-DD intervention.

People with T2DM have a 24% higher risk of depression than healthy people, and people developing depression are more likely to develop T2DM [10, 11]. Patients with depression often have poor compliance, which in turn worsens glycemic control and life quality, further aggravating the progression of T2DM [12]. Therefore, it is crucial to provide an effective treatment to such patients with symptoms of depression.

Abnormal expression of RNA during transcription is involved in the pathogenesis of DD [13, 14]. Long non-coding RNA (lncRNA) is an endogenous RNA molecule with a length of over 200 bp and limited protein-coding ability [15]. Circular RNAs (circRNAs) are a group of ring RNA molecules which are generally composed of more than one exon [16]. Recent studies have shown that lncRNAs and circRNAs are closely related to DD and suggest that may be promising targets for its treatment [15–17]. However, whether Baduanjin plays a role as an anti-DD intervention in association with lncRNA and circRNA remains unclear. Therefore, in this study, we aimed to investigate the hypoglycemic effect and the reduction in depression index due to Baduanjin, as well as its effects on the expression of lncRNAs and circRNAs, in patients with DD.

## Materials and methods

The Minimum Standards of Reporting Checklist contains details of the experimental design, and statistics, and resources used in this study (Additional file 1).

### Experimental objects and grouping

This study was approved by the Ethics Committee of Beijing University of Chinese Medicine (BUCM) (2016BZHYLL0105) and conducted in accordance with the principles of the Declaration of Helsinki. All enrolled patients were from hospitalized patients at the BUCM affiliated hospital (2016.1–2016.4) and were confirmed to have signed informed consent after enrollment. Cutoff points of 44 (total coarse points) and 55 (standard score) were used to define depression for the Self-Rating Depression Scale (SDS), whereas a score of 5 or more was used as a cutoff to define depression for patient health questionnaire 9 (PHQ9). We performed a 12-week Baduanjin intervention on DD patients enrolled in the study (starting at 8:30 am every day for approximately 30 min). Fasting blood samples of patients in the DD group were collected before and after the intervention of Baduanjin for further experiments. More specifically speaking, at 8:00 am on the 0th and 12th week of the intervention, the patient was in a state of peace and fasting, and blood was collected by nurse.

### RNA extraction and library construction

Total RNA was extracted from whole blood according to a previously reported method [18]. Briefly, purification was performed using TRIzol reagent (Invitrogen, Carlsbad, CA, USA) and with the RNeasy Mini Kit (Qiagen, Hilden, Germany) according to the manufacturers protocol. Total RNA samples were then subjected to agarose electrophoresis and Nanodrop quality control and quantification. Enrich mRNA with oligo (dT) beads (rRNA removal kit for RNA degradation or prokaryotic); RNA sequencing library are completed by the KAPA Stranded RNA-Seq Library Prep Kit (Illumina). Finally, the constructed library was checked with the Agilent 2100 (NanoDrop ND-1000) and quantified by qPCR.

### Illumina sequencing

Sequencing libraries of well-pooled different samples were denatured by 0.1 M NaOH to generate single-stranded DNA, which was in situ expanded on TruSeq SR Cluster Kit v3-cBot-HS (#GD-401-3001, Illumina) after dilution to 8 pM concentration The resulting fragment ends are sequenced using a Sequencer such as the Illumina HiSeq 4000 for 150 cycles.

### Quantitative analysis of genes and circRNAs expression

Transcript abundance calculations were performed by software StringTie by aligning the results to known transcriptomes and then calculated by Ballgown. The units of expression were expressed as FPKM (Fragments Per Kilobase of gene/transcript model per Million mapped fragments). Genes were expressed at a threshold of FPKM ≥ 0.5 and the average number of genes with FPKM exceeding 0.5 in each group was considered to be expressed in the subgroup and statistically analyzed.

The expression of circRNA (Backsplice junction reads count) was quantified by the software STAR and then compared to the known transcriptome for junction locus detection and then calculated by CIRCexplorer2 for Backsplice junction reads. Unlike genes, the circRNA expression threshold is that CircRNAs with mean CPM exceeding 100 in each group are considered as expressed in the group and statistically analyzed.

### qRT-PCR

qRT-PCR was performed to verify the expressions of lncRNAs and mRNAs related to the occurrence and development of DD. Purified total RNAs isolated from 14 samples were reverse-transcribed into cDNA according to the manufacturer’s instructions. Data were analyzed using the 2^−△△CT^ relative quantification method and normalized to β-actin to calculate relative lncRNA and mRNA concentrations. All primers are shown in Table 1.

**Table 1:** mRNA and lncRNA primers for quantitative PCR analysis

Primer name	Sequence
β-actin (H)	F: “5′ GTGGCCGAGGACTTTGATTG 3′” R :“5′ CCTGTAACAACGCATCTCATATT3′”
CXCL8	F: “5′ CATACTCCAAACCTTTCCACC 3′” R : “5′ ACTTCTCCACAACCCTCTGC 3′”
CXCR4	F: “5′ ATAAAATCTTCCTGCCCACC 3′ R : “5′ TACTTGTCCGTCATGCTTCTC3′”
DUSP2	F: “5′ TTTTCCGCTACAAGAGTATCCC 3′” R : “5′ CACCCAGTCAATGAAGCCTAT 3′”
IL1B	F: “5′ CCGACCACCACTACAGCAAG 3′” R : “5′ TGGACCAGACATCACCAAGC 3′”
NR4A2	F: “5′ CAGTGGAGGGTAAACTCATCTT 3′” R : “5′ TTCCTTGAGCCCGTGTCT 3′”
OSM	F: “5′ CTGCTCGAAAGAGTACCGC 3′” R : “5′ GCCCAGTGTGGCATTGAG 3′”
NEAT1	F: “5′GCTGCATCTTCTAAATTGAGCC 3′” R: “5′GCAAACAGGTGGGTAGGTGAG 3′”

### Gene Ontology and KEGG pathway analysis

We used software topGO for Gene Ontology (GO) analysis to obtain significantly enriched GO articles and corresponding genes to deduce important biological functions involved in differentially expressed genes. Meanwhile, based on the newest KEGG database (Kyoto Encyclopedia of Genes and Genomes), find out the pathways which are most associated with differentially expressed genes.

### Gene set enrichment analysis (GSEA)

GSEA is a computational method used to determine whether a given gene set has significant differences among different groups [19]. Genes in these sets have some degree of correlation. Therefore, enrichment analysis of gene sets can make up for the shortcomings of single gene in the analysis.

### Correlation and co-expression analysis

An lncRNA-mRNA regulatory network was constructed based on the inter-regulatory association between differentially expressed genes in the blood of DD patients following the Baduanjin intervention.

### Statistical analysis

The results are expressed as mean ± standard error (SEM). GO and pathway enrichment analysis was done using Fisher’s test, the smaller the *P* value the greater the contact between the GO and pathway terms and the input differentially expressed genes. Two-sided P values < 0.05 were considered statistically significant.

## Results

### Hypoglycemic effect and reduction in depression index due to Baduanjin

**Fig. 1:**
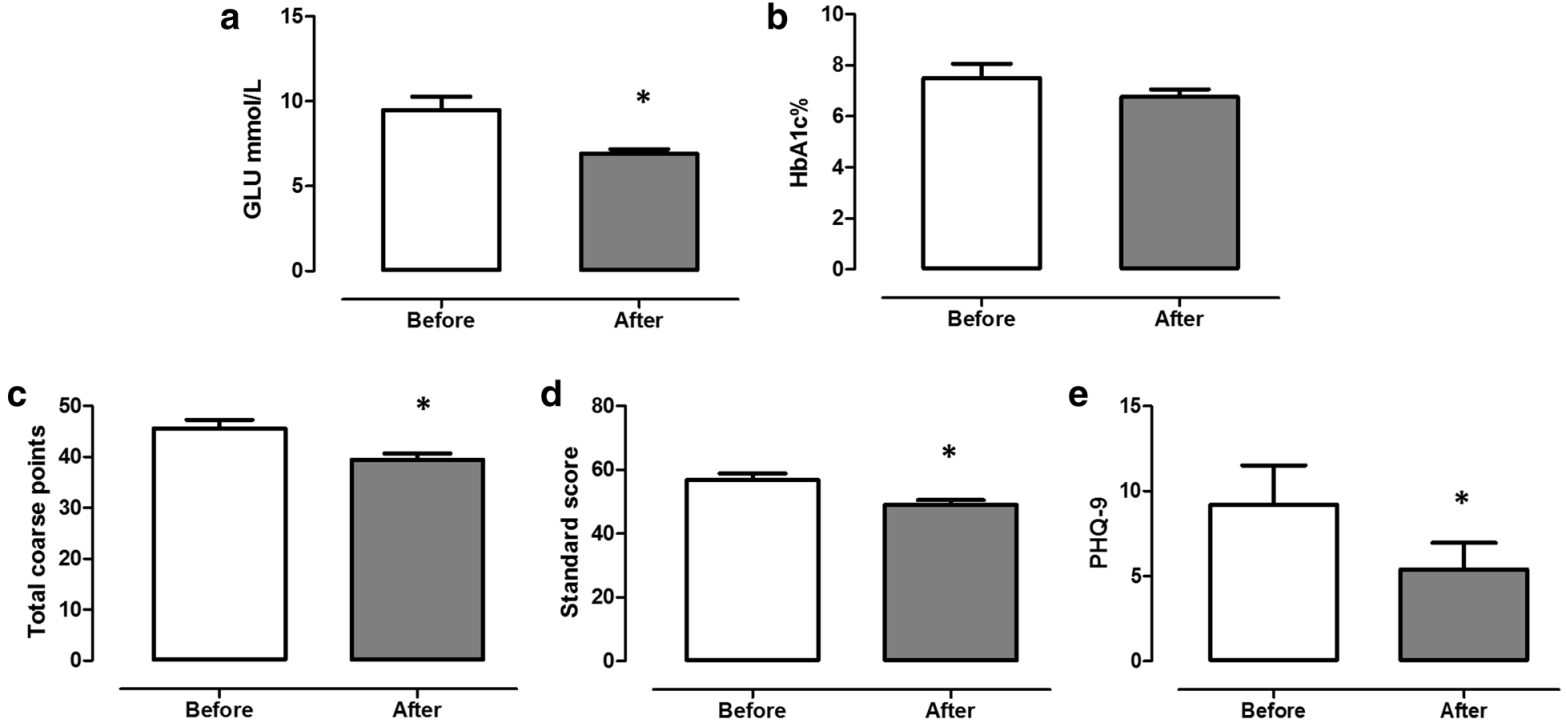
Effects of Baduanjin on GLU, HbA1c % and depression index in DD patients. Data are expressed as mean ± SEM. *p < 0.05 compared with BDD, n = 5

We compared fasting blood glucose levels (GLU), HbA1c %, SDS, and PHQ9 in patients with DD before and after the Baduanjin intervention. The results showed that after the intervention of Baduanjin (ADD), HbA1c % did not significantly differ than that before Baduanjin intervention (BDD). However, the levels of fasting blood glucose, PHQ9, total coarse points, and standard score of ADD were significantly lower than those of BDD (Fig. 1 and Table 2).

**Table 2:** Effects of Baduanjin on GLU and depression coefficient in DD patients

			Before-Baduanjin interventions					After-Baduanjin interventions				
ID	Age	Sex	Total coarse points	Standard score	PHQ-9	GLU	HbA1c %	Total coarse points	Standard score	PHQ-9	GLU	HbA1c %
206	60	Female	49	61	17	8.2	7	40	50	6	6.3	6.4
103	48	Female	46	57	6	10.2	6.9	42	52	11	7.3	6.5
205	62	Male	44	55	5	12.2	9.7	36	45	2	9.5	7.9
117	55	Female	49	61	12	7.9	7.3	42	52	5	6.5	6.8
104	54	Female	40	50	6	8.9	6.6	37	46	3	6.6	6.2

### Effect of Baduanjin on mRNA and circRNA expression in patients with DD

**Fig. 2:**
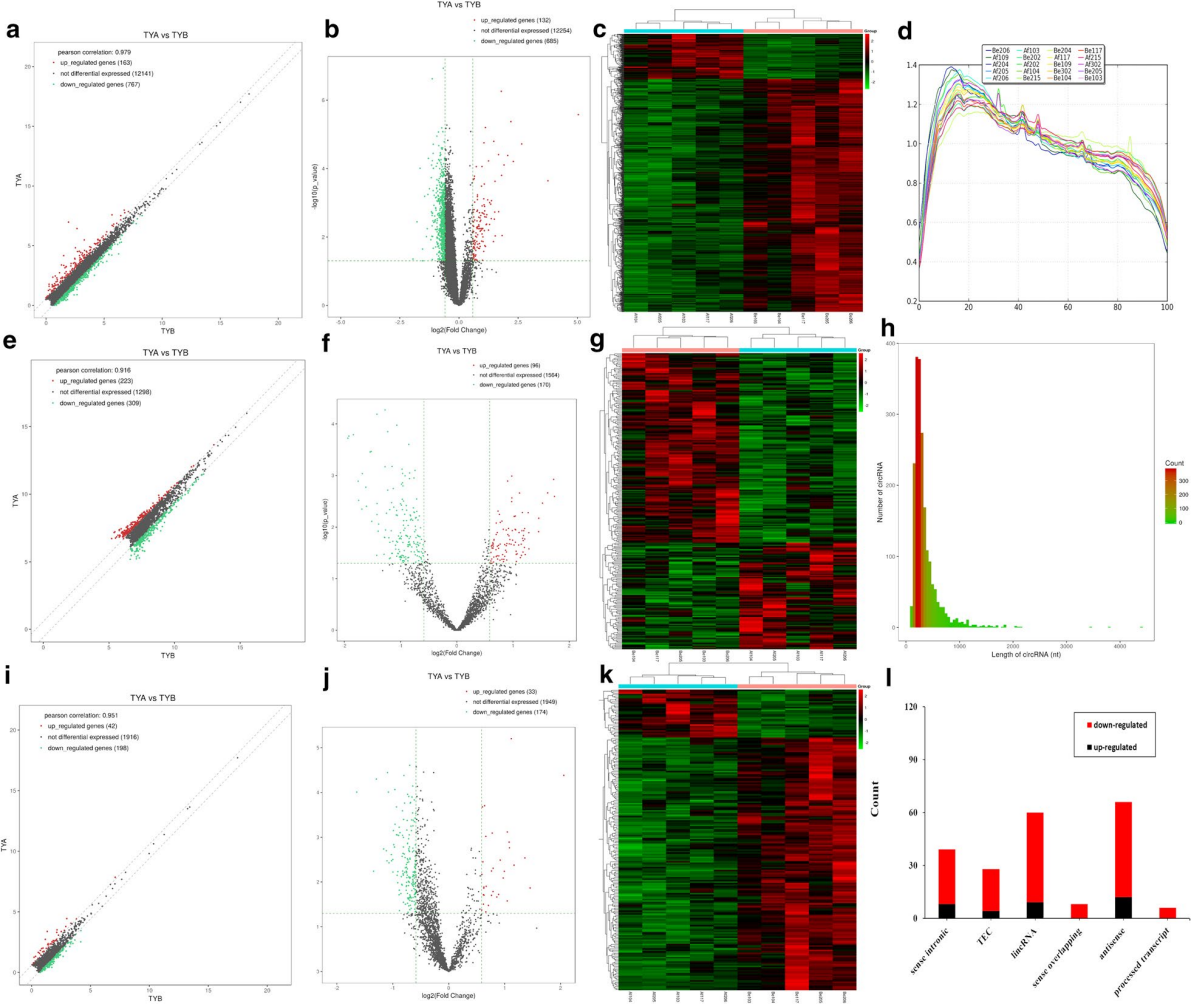
Volcano-Plot, Scatter plot and Hierarchical clustering of differentially expressed mRNAs (**a**, **b**, **c**), circRNAs (**e**, **f**, **g**) and lncRNAs (**i**, **j**, **k**). Volcano-Plot above the green parallel line (P < 0.05) and outside the two longitudinal green lines indicated differentially expressed genes between the two compared samples. Scatter plot x-axis and y-axis represent FPKM mean values (log2 transitions) of two groups of gene. Analogous, green and red dots represent down-and up-regulated genes respectively, and the gray dots represent non-significantly different genes. **d** The transcriptional coverage profile is used to assess the 5 ‘or 3’ preference of the sample library fragment. **h** circRNA length distribution chart. X-axis and y-axis represent the number and the longth of circRNAs, respectively. **i** Types and counts of differentially regulated lncRNAs detected by Illumina sequencing (log2FC ≥ 0.585 and P < 0.05). The amount of each lncRNA type was accumulated in a bar graph, showing up-regulated and down-regulated lncRNAs, respectively

Compared to BDD, 610 (99 up-regulated and 511 down-regulated) and 266 (170 down-regulated and 96 up-regulated) differentially expressed mRNAs and circRNAs were identified in ADD, respectively (P < 0.05 and fold change > 1.5; Additional file 2: Table S1 and Additional file 3: Table S2). The scatterplot and the volcano plot of the differentially expressed mRNAs and circRNAs are shown in Fig. 2a–h. In addition, the fragment distribution of each sample is balanced and concentrated within 15 to 80 bp, indicating that the file construction quality is good (Fig. 2d).

### Effect of Baduanjin on the expression of lncRNAs in patients with DD

We detected 33 up-regulated and 174 down-regulated lncRNAs in ADD as compared to BDD (P ≤ 0.05, log2FC ≥ 0.585; Additional file 4: Table S3). According to the relative positions of lncRNAs and protein-coding genes in the genome, we classified these differentially expressed lncRNAs into 6 categories (Fig. 2i–l). In our data, the number of antisense lncRNAs was the most, in which 12 were up-regulated and 54 were down-regulated.

### Effect of Baduanjin on DD-related antisense lncRNAs and their corresponding mRNA

Antisense lncRNA regulates the expression of the corresponding sense mRNA by inducing epigenetic changes in DNA [20]. Therefore, in the current study, we integrated differentially expressed antisense-lncRNAs and their differentially expressed mRNAs to deduce the function of the lncRNAs. We found a total of 153 corresponding sense mRNAs, 33 of which were up-regulated and 120 were down-regulated. In addition, 41 of these antisense lncRNAs were intronic antisense and 112 were natural antisense. As shown in Table 3, we listed the top 20 differentially expressed antisense LncRNAs based on their log2FC values. Furthermore, we also defined highly conserved, tissue-specific, disease-related, and biological processes-related lncRNAs.

**Table 3:** Co-analysis of differentially expressed antisense lncRNAs and mRNAs

lncRNA ID	log2FC	p value	Relationship	mRNA ID	log2FC	p value
*Up*-*regulated*						
ENST00000620937	2.058	0.000	antisense	ENST00000314028.10_1	0.410	0.473
ENST00000620937	2.058	0.000	antisense	ENST00000392334.6_1	− 0.141	0.516
ENST00000492398	1.715	0.000	antisense	ENST00000339355.2_1	0.286	0.092
ENST00000465443	1.265	0.003	antisense	ENST00000339355.2_1	0.286	0.092
ENST00000443093	1.119	0.000	antisense	ENST00000407797.5_1	0.044	0.834
ENST00000623876	1.100	0.004	antisense	ENST00000262027.9_1	− 0.371	0.385
ENST00000623876	1.100	0.004	antisense	ENST00000547665.5_1	− 0.175	0.417
ENST00000623876	1.100	0.004	antisense	ENST00000552914.5_2	0.384	0.006
ENST00000623876	1.100	0.004	antisense	ENST00000628866.2_1	0.065	0.901
ENST00000606596	1.086	0.002	antisense	ENST00000256255.10_2	− 0.079	0.761
ENST00000606596	1.086	0.002	antisense	ENST00000521265.5_2	− 0.583	0.256
ENST00000606596	1.086	0.002	antisense	ENST00000545648.2_1	− 0.117	0.638
ENST00000419808	0.990	0.002	antisense	ENST00000264161.8_1	− 0.500	0.104
ENST00000586483	0.882	0.029	antisense	ENST00000588173.1_1	0.458	0.228
ENST00000444406	0.816	0.001	antisense	ENST00000264161.8_1	− 0.500	0.104
*Down*-*regulated*						
ENST00000587498	− 1.799	0.038	antisense	ENST00000255608.8_1	− 0.520	0.084
ENST00000542598	− 1.164	0.002	antisense	ENST00000064778.8_1	− 0.178	0.263
ENST00000542598	− 1.164	0.002	antisense	ENST00000356467.4_1	0.386	0.114
ENST00000492808	− 1.079	0.003	antisense	ENST00000374217.6_1	− 0.177	0.689
ENST00000492808	− 1.079	0.003	antisense	ENST00000374222.5_1	− 0.207	0.564
ENST00000564919	− 1.068	0.004	antisense	ENST00000396593.6_2	− 0.093	0.879
ENST00000564919	− 1.068	0.004	antisense	ENST00000611932.4_2	0.331	0.405
ENST00000564919	− 1.068	0.004	antisense	ENST00000618335.4_2	0.046	0.932
ENST00000564919	− 1.068	0.004	antisense	ENST00000619881.4_2	− 0.512	0.281
ENST00000544289	− 1.065	0.000	antisense	ENST00000361716.7_1	− 0.925	0.000
ENST00000544289	− 1.065	0.000	antisense	ENST00000400911.7_1	0.330	0.345
ENST00000621891	− 1.005	0.001	antisense	ENST00000336095.10_1	0.100	0.767
ENST00000425081	− 0.983	0.001	antisense	ENST00000313486.11_1	− 0.283	0.567
ENST00000425081	− 0.983	0.001	antisense	ENST00000539476.5_1	− 0.031	0.972
ENST00000458296	− 0.944	0.001	antisense	ENST00000354258.4_1	− 0.278	0.208

### Enrichment analysis of differentially expressed genes

**Fig. 3:**
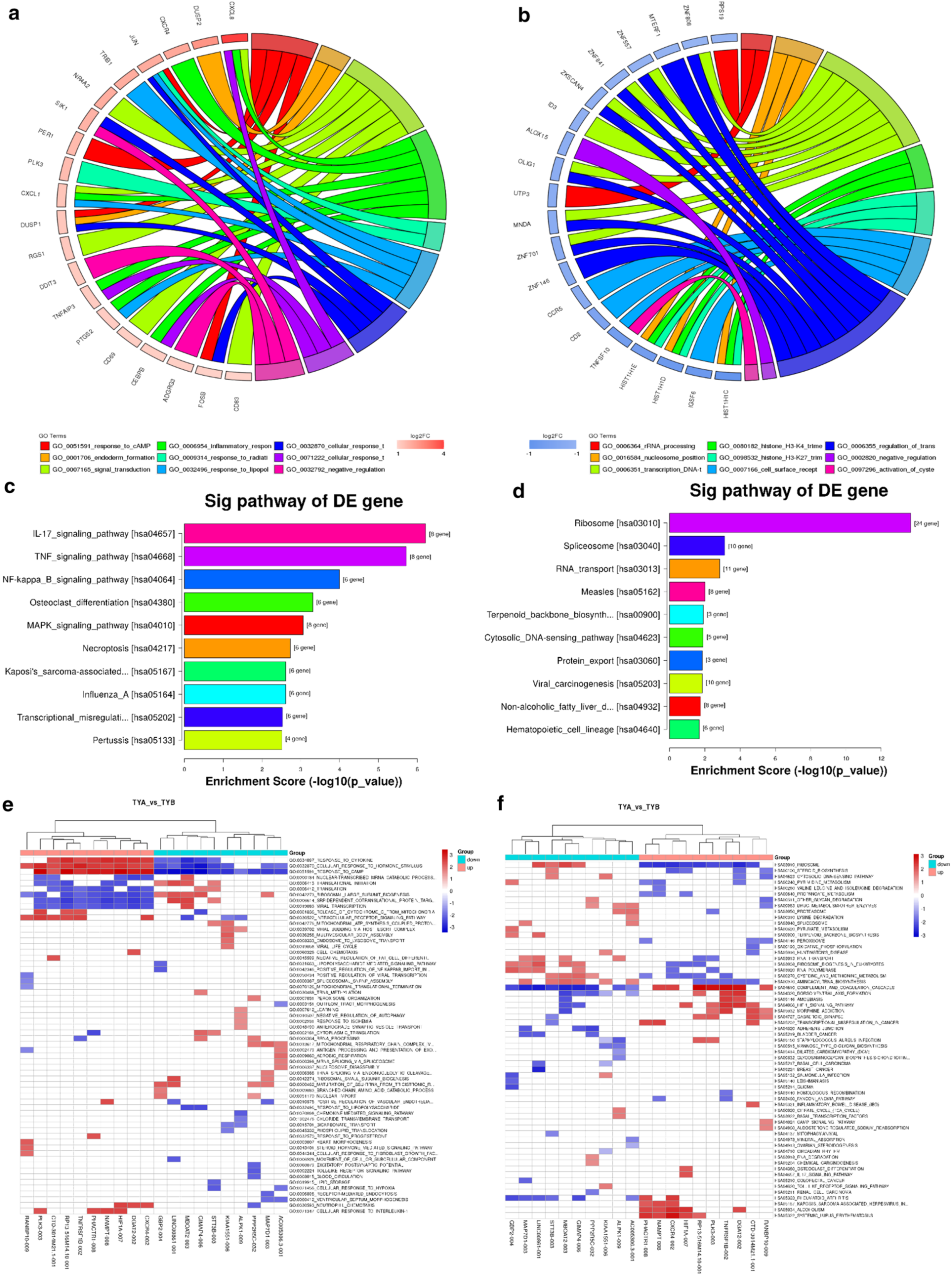
Circle drawing represented the mainly involved BP of up-regulated (**a**) and down-regulated (**b**) differentially expressed genes. Bar chart of the top ten entries KEGG pathway analysis up (**c**) and down (**d**). Sorts from low to high according to P-values and the abscissa indicates enrichment score (− log10 (P value)). Difference lncRNAs GSEA Cluster Heat Map. (**e**) Biological Process and (**f**) KEGG Pathway, each row represents a functional entry, and each column represents an lncRNA

GO analysis revealed the functional effects of Baduanjin on differentially expressed mRNAs in blood samples of patients with DD. Our results showed that up-regulated mRNAs were associated with biological processes such as inflammatory reactions, while the down-regulated mRNAs were associated with immune-related processes. Interestingly, the inflammatory response is closely related to depression, and it can play an important role in controlling the symptoms of depression (Fig. 3a, b).

Pathway analysis of differentially expressed genes is devised to comprehend pathways and molecular interactions of the related mRNAs. The results showed that a total of 22 pathways were associated with the up-regulated mRNAs and 15 to the down-regulated mRNAs. Of the up-regulated protein-coding mRNAs, the highest number of mRNAs coded proteins involved in the IL-17 and TNF signaling pathways, whereas, the highest number of down-regulated mRNAs were related to the ribosome and spliceosome machineries (Fig. 3c, d). Hence, it is suggestive that these pathways might play a role in this intervention.

### GSEA analysis of differentially expressed lncRNAs

In order to more intuitively understand the pathways and biological processes the differentially expressed lncRNAs are involved in, we used a powerful analytical method called GSEA to interpret lncRNA expression data (Fig. 3e, f). Compared with BDD, we found that the differentially expressed lncRNAs in the blood of ADD were mainly associated with biological processes such as response to cytokines, cellular response to hormonal stimuli, response to CAMP, positive regulation of NF-ƙB, and pathways related to ribosome machinery, cytosolic DNA-sensing pathway, and steroid biosynthesis. Therefore, the lncRNA-GSEA makes up for the deficiencies of the single lncRNA in the analysis, providing insights for the next in-depth study of the role of Baduanjin in decreasing the symptoms of DD.

### Effect of Baduanjin on lncRNA-mRNA regulatory network

**Fig. 4:**
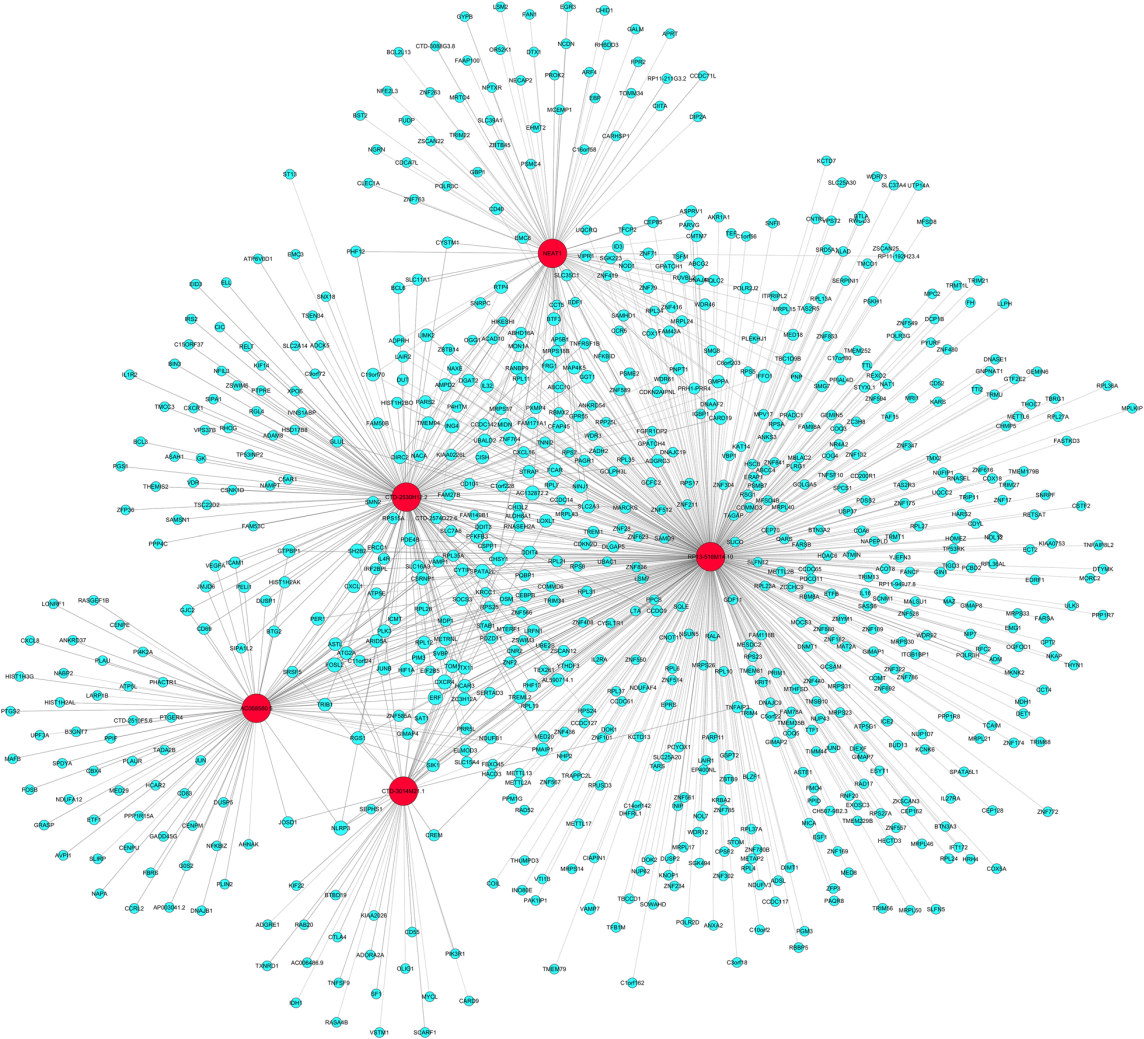
LncRNA-mRNA regulatory network. Red and blue circles represent lncRNA and mRNA, respectively. The solid line is positively correlated and the dotted line is negatively correlated

We screened 5 up-regulated lncRNAs (RP13-516M14.10, NEAT1, CTD-2530H12.2, CTD-3014M21.1, and AC068580.5) to construct the lncRNA-mRNA regulatory network map. A total of 1045 related mRNA were identified, of which 688 were negatively correlated with the corresponding lncRNAs and 357 mRNAs were positively correlated with the corresponding lncRNAs (Fig. 4). Five of our differentially expressed mRNAs (CXCL8, DUSP2, OSM, CXCR4, and NR4A2) were identified in the above lncRNA-mRNA regulatory network and defined as the key nodes of the lncRNA-mRNA regulatory network.

## Discussion

**Fig. 5:**
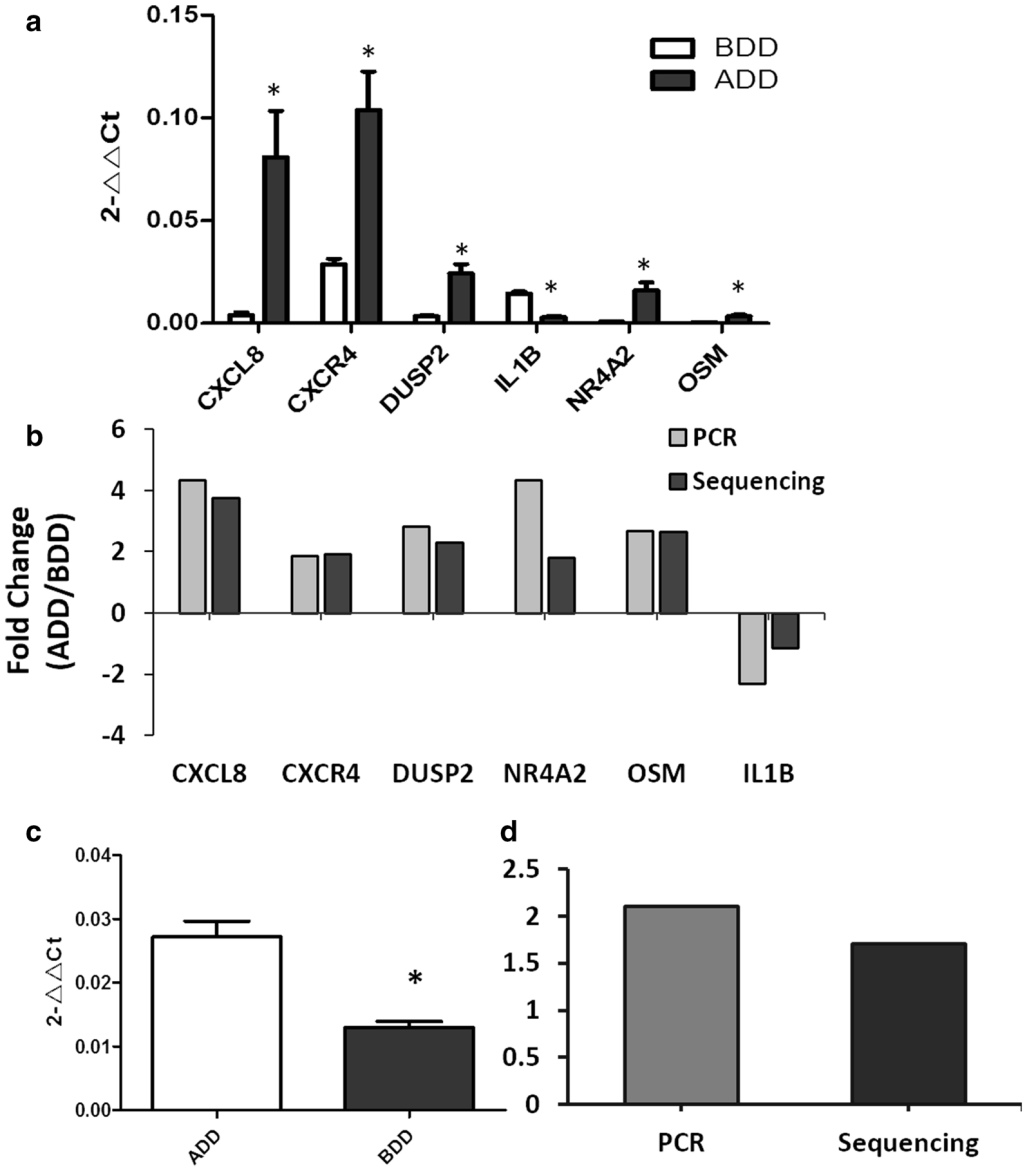
qRT-PCR validated data. **a** Comparison of differentially expressed mRNA qRT-PCR data, **b** Comparison qRT-PCR and Sequencing data. **c** Comparison of NEAT1 qRT-PCR data, **d** Comparison of the results of qRT-PCR and Sequencing.*P < 0.05 versus BDD group, N = 5

In this study, we found that Baduanjin intervention significantly reduced blood glucose levels, and lowered the depression coefficient in patients with DD, showing an anti-DD effect. Results of the current study are in line with a previous review indicating that physical activity has a positive effect on regulating mood and reducing the symptoms of depression [21]. 610 and 207 differentially expressed mRNAs and lncRNAs, respectively, were screened before and after Baduanjin intervention. Subsequently, we selected six differentially expressed mRNAs and 1 differentially expressed lncRNA for RT-PCR validation, confirming the reliability of our sequencing results (Fig. 5). Our results revealed a 1.69 fold up-regulation in *lncRNA*- *nuclear enriched abundant transcript 1 (NEAT1)* in BDD group, which is consistent with previous studies [22, 23]. It has been known that NEAT1 is a competitive endogenous RNA, and it was closely related to T2DM, neurodegenerative diseases, epilepsy, and other mental illnesses [24–26].

The disks large-associated protein (DLGAP) family serves as a scaffold in postsynaptic density, which can link the glutamate receptors in the postsynaptic membrane with other glutamate receptors, signaling proteins, and cytoskeletal components [27]. With the central localization in the postsynapse, the DLGAP family plays a significant role in synaptic scaling by adjusting the turnover of both ionotropic and metabotropic glutamate receptors in response to synaptic activity [28]. Therefore, the DLGAP family is involved in the development of a variety of psychological and neurological diseases. As a member of the DLGAP family, DLGAP1-AS1 is expected to become a molecular target for the treatment of DD. Compared with BDD, we found that *lncRNA*-*DLGAP1*-*AS1* was down-regulated in ADD. This shows that Baduanjin may play a role by down-regulating DLGAP1-AS1.

Since most of the differentially expressed lncRNAs have not been studied, we have constructed mRNA expression profiles to help study the effect of Baduanjin on DD. Depression can be perceived as a psycho-neuro-immunological disorder and the chemokine system plays a crucial role in its pathogenesis [29–32]. In this study, we also found increased expression of two chemokines (CXCL8 and CXCR4) after Baduanjin intervention. OSM are associated with depression [33]. NR4A2 (Nur-related receptor 2), is an orphan nuclear receptor that can be constitutively active as a transcription factor and can influence the expression of genes important for human brain development and regulation [34]. In our results, compared with BDD, the expression level of NR4A2 was significantly up-regulated in ADD (FD = 3.448, P < 0.05), suggesting that the anti-DD effect of the Baduanjin may be exerted through the regulation of the expression of NR4A2.

KEGG pathway analysis revealed that Baduanjin may play a pivotal role in DD progression through the significantly enriched 20 pathways including IL-17 and TNF. Our results support previous discovery [35, 36]. Studies have shown that IL-17 pathway is closely related to severe depression and depression can inhibit the activity of the IL-17 pathway [37, 38]. Our study found that the activity of the IL-17 pathway was significantly increased after Baduanjin intervention in patients with DD, suggesting that Baduanjin exerts anti-DD effects by up-regulating the IL-17 pathway.

The improvement of the depression index and blood glucose levels in patients with DD by Baduanjin intervention was significantly associated with the significant enrichment of GO processes in the blood of these patients. According to the annotation results, the most significant GO processes are cellular response to lipopolysaccharide, nuclear-transcribed mRNA catabolic process nonsense-mediated decay, and inflammatory response, indicating that the related coding genes contributed to development of DD. Interestingly, the top up-regulated biological processes belong to the immune pathway, which is closely related to depression [39]. Therefore, it is further confirmed that the Baduanjin intervention can improve the immune system function which is related to the anti-DD effect.

In addition, five significant differentially expression mRNAs (CXCL8, DUSP2, OSM, CXCR4, and NR4A2) were identified in the lncRNA-mRNA regulatory network and defined as the key nodes of the network. Among them, differentially expressed mRNA-CXCL8 was positively correlated with AC068580.5, DUSP2 was positively correlated with RP13-516M14.10, mRNA-OSM was positively correlated with RP13-516M14.10, AC068580.5, and CTD-2530H12.2, mRNA-CXCR4 was positively correlated with RP13-516M14.10, CTD-3014M21.1, AC068580.5, and CTD-2530H12.2, and mRNA-NR4A2 was positively correlated with RP13-516M14.10. The lncRNA-mRNA regulatory network analysis confirmed the crosslinking and complex regulation patterns of lncRNA and mRNA expression and verified the authenticity of the regulatory network. DD-related lncRNA-mRNA regulatory network and the exploration of their regulatory relationships have provided a molecular basis for the study for the treatment of DD with Baduanjin.

## Conclusions

To conclude, our research is the first systematic profiling of mRNA, lncRNA, and circRNA in patients with DD undergoing Baduanjin intervention. We demonstrated that a 12-week Baduanjin intervention could significantly reduce blood glucose, SDS, and PHQ9 scale scores in patients with DD. These effects may be achieved by regulating the expression of the non-coding RNAs. This study hints towards an epigenetic role of Baduanjin intervention in DD and provides valuable insights in its potential mechanism.

## Additional files

**Additional file 1.** Minimum Standards of Reporting Checklist.

**Additional file 2.** Differentially expressed mRNAs.

**Additional file 3.** Differentially expressed circRNAs.

**Additional file 4.** Differentially expressed lncRNAs.

## Abbreviations

T2DM: type 2 diabetes
DD: T2DM with depression
ADD: after Baduanjin intervention group
BDD: before Baduanjin intervention
GO: Gene Ontology
RT-PCR: reverse transcription-polymerase chain reaction
lncRNAs: long non-coding RNAs
circRNAs: circular RNAs
KEGG: Kyoto Encyclopedia of Genes and Genomes
GSEA: gene set enrichment analysis
GLU: glucose
SDS: Self-Rating Depression Scale
PHQ9: patient health questionnaire 9
NEAT1: nuclear enriched abundant transcript 1
DLGAP: discs large associated protein
NR4A2: Nur-related receptor 2
